# Survival rates in the world's southernmost forest bird community

**DOI:** 10.1002/ece3.10143

**Published:** 2023-06-20

**Authors:** Erik M. Sandvig, Claudio S. Quilodrán, Tomás A. Altamirano, Francisco Aguirre, Omar Barroso, Juan Rivero de Aguilar, Michael Schaub, Marc Kéry, Rodrigo A. Vásquez, Ricardo Rozzi

**Affiliations:** ^1^ Cape Horn International Center Universidad de Magallanes Puerto Williams Chile; ^2^ Centro Bahía Lomas, Facultad de Ciencias Universidad Santo Tomás Santiago Chile; ^3^ Swiss Ornithological Institute (Vogelwarte) Sempach Switzerland; ^4^ Departamento de Ciencias Ecológicas, Facultad de Ciencias, Instituto de Ecología y Biodiversidad (IEB) Universidad de Chile Santiago Chile; ^5^ Department of Genetics and Evolution University of Geneva Geneva Switzerland; ^6^ Audubon Americas, National Audubon Society Santiago Chile; ^7^ Center for Local Development (CEDEL), Villarrica Campus Pontificia Universidad Católica de Chile Villarrica Chile; ^8^ Centro de Investigación Gaia Antártica (CIGA) Universidad de Magallanes Punta Arenas Chile; ^9^ Sub‐Antarctic Biocultural Conservation Program, Department of Philosophy and Religion and Department of Biological Sciencies University of North Texas Denton Texas USA; ^10^ Department of Biological Sciences University of North Texas Denton Texas USA

**Keywords:** capture–mark–recapture, climate change, forest birds, hierarchical model, multispecies CJS model, Patagonia, survival

## Abstract

The Magellanic sub‐Antarctic Forest is home to the world's southernmost avian community and is the only Southern Hemisphere analogue to Northern Hemisphere temperate forests at this latitude. This region is considered among the few remaining pristine areas of the world, and shifts in environmental conditions are predominantly driven by climate variability. Thus, understanding climate‐driven demographic processes is critical for addressing conservation issues in this system under future climate change scenarios. Here, we describe annual survival patterns and their association with climate variables using a 20‐year mark–recapture data set of five forest bird species in the Cape Horn Biosphere Reserve. We develop a multispecies hierarchical survival model to jointly explore age‐dependent survival probabilities at the community and species levels in a group of five forest passerines. At the community level, we assess the association of migratory behavior and body size with survival, and at the species level, we investigate the influence of local and regional climatic variables on temporal variations of survival. We found a positive effect of precipitation and a negative effect of El Niño Southern Oscillation on juvenile survival in the white‐crested Elaenia and a consistent but uncertain negative effect of temperature on survival in juveniles and 80% of adults. We found only a weak association of climate variables with survival across species in the community and no temporal trends in survival for any of the species in either age class, highlighting apparent stability in these high austral latitude forests. Finally, our findings provide an important resource of survival probabilities, a necessary input for assessing potential impacts of global climate change in this unique region of the world.

## Graphical abstract

In this article, we describe the temporal dynamics in survival probabilities for the world's southernmost forest bird community and their relationship to climatic variables. We develop a multispecies hierarchical survival model using Bayesian inference to jointly explore age‐dependent survival probabilities at the community and species levels in a group of nine forest passerines using a 20‐year mark–recapture data set from the Cape Horn Biosphere Reserve, Chile. We find that winter precipitation and El Niño Southern Oscillation are good predictors of juvenile survival in two species; however, no temporal trends in survival for any species as juveniles or adults were found. Our results provide important baseline data and novel demographic information for several species in an understudied corner of the world.

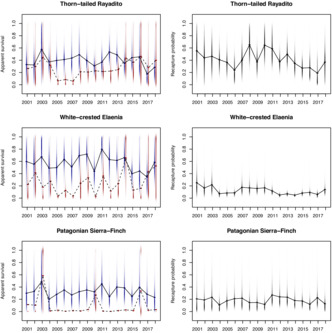

## 1 INTRODUCTION

Climate change poses a significant threat to the persistence of bird populations (Pearce‐Higgins et al., 2014; Wormworth & Sekercioglu, 2011). Evidence of its impact on population dynamics is widespread (Jenouvrier, 2013; Sæther & Engen, 2010; Scridel et al., 2018), yet our current understanding of the underlying mechanisms in temperate forest bird populations comes mainly from the Northern Hemisphere (Desante & Saracco, 2021; Julliard et al., 2004; Stephens et al., 2016; Virkkala & Rajasärkkä, 2011). The extent population dynamics respond to climate change varies geographically (Virkkala & Lehikoinen, 2014) and depends on the life‐history characteristics of a species (Jiguet et al., 2010). Critically, the demographic information required to predict population outcomes under future climate conditions is still lacking in many areas of the world, which limits the ability to make informed conservation decisions regarding the management of species and their habitats across the globe. The Magellanic sub‐Antarctic region in southern Patagonia is considered one of the few remaining pristine areas of the world (Mittermeier et al., 2003), where direct anthropogenic pressures are minimal and environmental change is thought to be mainly influenced by climate change, making it a promising area to study climate–demography relationships.

Temporal changes in survival are an important component of population dynamics because they can ultimately translate to changes in population growth rates (Saracco & Rubenstein, 2020; Woodworth et al., 2017). Survival can vary widely between years as a result of variations in environmental conditions (Dybala, Eadie, et al., 2013), such as temperature and/or precipitation. Pearce‐Higgins et al. (2014) reviewed the relationship between local weather and survival in birds, with many studies observing a positive association with increasing temperatures, particularly at higher latitudes. In contrast, they found less consistency for the effects of precipitation, with negative associations at lower latitudes but positive at higher latitudes. When looking at these effects, it is also important to consider the species' life history. The climatic conditions during the nonbreeding season are thought to be one of the main determinants of interannual survival (Pearce‐Higgins et al., 2014), where adverse weather during winter, namely low temperatures, snow cover, heavy precipitation, and frost can negatively impact the species' survival rates (Dybala, Eadie, et al., 2013; Robinson et al., 2007).

Indices for broad‐scale climate patterns, such as the El Niño Southern Oscillation (ENSO) index, simplify an aggregate of climatic components (e.g., temperature, precipitation, winds) acting over large geographical areas. They are often used to assess the impact of climate on avian population dynamics (Nott et al., 2002; Post & Forchhammer, 2002; Stenseth et al., 2003), as they also influence temperature and precipitation patterns at a local scale. Although ENSO has shown to be an important predictor of variation in survival, the magnitude and directionality of its impacts widely vary across space (Jenouvrier, 2013; Sillett et al., 2000). Thus, the mechanisms by which it acts at a local scale are often poorly understood but likely vary among ecological communities (Wan et al., 2022).

In addition to considering ‘where’ (e.g., latitude) and ‘when’ (e.g., season) weather is most impactful to survival, it is also important to consider the species' migratory behavior. Migratory species are particularly susceptible to the effects of climate change (Møller et al., 2008). This is because asynchronies between food abundance and arrival dates on breeding territories (Simmonds et al., 2020), or shifts in precipitation patterns on the wintering grounds (Schaub et al., 2005), can negatively impact survival. In addition, survival generally differs between age classes (Fay et al., 2015; Pizarro Muñoz et al., 2018; Sandvig et al., 2017), with survival in the early stages of life (juveniles) being most sensitive to weather compared with adults (Dybala, Gardali, & Eadie, 2013; Oro et al., 2010; Robinson et al., 2007). This is an important consideration for predicting population‐level effects of environmental change (Dybala, Eadie, et al., 2013) because the differing sensitivities to climate between age classes can influence their contributions to population growth rates (Finkelstein et al., 2010).

Life‐history theory postulates that adult survival should be lower in temperate regions compared with the tropics, due to higher productivity in the latter (Roff, 2002). Survival in the Northern Hemisphere generally decreases with latitude (Pizarro Muñoz et al., 2018; Scholer et al., 2020); however, there is mixed support for this gradient in the Southern Hemisphere. Latitudinal gradients in ecology are often generalized to be equal between hemispheres in spite of hemispheric asymmetries due to contrasting environmental conditions (Chown et al., 2004). An extensive review found that although there was no evidence of a latitudinal survival gradient in the Southern Hemisphere as a whole, there was evidence of one in South America (Scholer et al., 2020). As many aspects of ecology and evolution in birds of the Southern Hemisphere have turned out to be different from those in the Northern Hemisphere (Ojeda et al., 2021; Theuerkauf et al., 2022), it is important to fill geographic gaps of information to assess the generality of these patterns between hemispheres. In contrast to similar latitudes in the Northern Hemisphere, the climate in the sub‐Antarctic region of South America is oceanic‐driven (oceans cover 98% of the latitudinal band surface), making seasonal temperature variation much less pronounced than in the Northern Hemisphere (Lawford et al., 1996). These conditions could potentially give rise to differing ecological patterns at similar latitudes between hemispheres (Martin et al., 2021).

The Magellanic sub‐Antarctic region in southern Chile and Argentina is home to the world's southernmost forests and is the only Southern Hemisphere analogue to Northern Hemisphere temperate forests at this latitude (Rozzi et al., 2012). The avian community is the most diverse vertebrate group in these forests (Rozzi & Jiménez, 2014), making them ideal candidates to study the potential effects of climate on vital rates across species. However, many aspects of the ecology of its bird community remain severely understudied. Demographic parameters have been estimated for less than 5% of Neotropical bird species (Ruiz‐Gutiérrez et al., 2012), and the Patagonian bird community is not an exception to this extreme paucity. Obtaining information on demographic processes in this region is an important component needed for adequately exploring potential conservation issues related to climate change.

In this study, we explore age‐dependent survival probabilities of passerines inhabiting the Magellanic sub‐Antarctic Forest. We investigate patterns both at the community and species level in a group of five species, using a 20‐year capture–recapture data set from the Cape Horn Biosphere Reserve (CHBR) in Chile. We aim to assess the association of survival probabilities with phenotypic and behavioral traits at the community level and whether they show temporal trends and how they are influenced by climatic variables at the species level. We also compare our survival estimates with those of conspecifics in other studies at different latitudes to assess the evidence for a latitudinal gradient in South America.

## 2 METHODS

### 2.1 Study site

Our study site is located within the CHBR, in the sub‐Antarctic Magellanic region (Rozzi & Jiménez, 2014), at the very southern tip of the South American continent. Data were collected at Omora Ethnobotanical Park banding station (54°57′S; 67°39′W), located 3 km west of the town of Puerto Williams, on the north coast of Navarino Island. This is the southernmost continental Long‐Term Ecological Research (LTER) site (Rozzi et al., 2020), with no equivalent in the Southern Hemisphere. The predominant habitats at the banding station are mixed *Nothofagus betuloides* and *Nothofagus pumilio* forest with *Drymis winteri* understory, and forest edge scrubland dominated by *Embothrium coccineum*, *Chilotrichium difussum*, *Berberis buxifolia*, and *Ribes magellanicum* (Rozzi & Jiménez, 2014). The mean annual precipitation recorded near the banding station is ~500 mm, and mean annual temperature is 6°C, ranging from 2°C in winter (July) to 10°C in summer (January; Rozzi & Jiménez, 2014).

### 2.2 Capture–recapture data

We used capture–recapture data from the LTER station designed to monitor forest birds. Standardized mist netting was used within the forest for 2 to 6 days each month of the year starting in 2000 (with few exceptions during this period). Here, we use only data collected during the breeding season (September to March; from 2001 to 2020) to remove possible transient individuals in species that form foraging flocks during winter. The monthly capture data were then collapsed to annual capture histories for estimating annual survival. Mist netting was done at two sites located roughly 500 m from each other. At each site, five 12‐m mist nets were placed individually, separated roughly 50 to 100 m between each mist net. Due to the similarity of the forest composition and proximity between the two sites, we consider them as a single site in the analysis. Among all 27 species with captures at the study site, we selected only those where adequate sample sizes were available, which we here defined to at least 50 captures and 4 recaptures. We finally selected five species to include in the model: thorn‐tailed Rayadito (*Aphrastura spinicauda*), white‐crested Elaenia (*Elaenia albiceps*), house wren (*Troglodytes aedon*), austral thrush (*Turdus falklandii*), and Patagonian sierra finch (*Phrygilus patagonicus*).

### 2.3 Climatic variables

To assess the association between weather and climatic conditions with the temporal dynamics of survival, we selected three climatic variables known to be important environmental predictors of survival (Dybala, Eadie, et al., 2013; Grosbois et al., 2008; Jenouvrier, 2013). For weather at the local scale, we used (a) daily minimum temperature and (b) daily precipitation. Because nonbreeding conditions are widely thought to be one of the main drivers of survival in passerines (Dybala, Eadie, et al., 2013; Robinson et al., 2007), we took the mean of the daily minimum temperature over the nonbreeding period (April to August) previous to the breeding season capture event, and the sum of daily precipitation over the same period. For climate at the regional scale, we used Multivariate ENSO Index. Temperature and precipitation data were obtained from the Guardiamarina Zañartu meteorological station, which is adjacent to the study site (54.9317°S; 67.6156°W) and managed by the Dirección Meteorológica de Chile. Missing weather data were filled‐in using the hourly data of the global reanalysis ERA5 for land (Copernicus Climate Change Service, 2017) and processed using Climate Data Operator (CDO) software (Schulzweida, 2019). The index values for ENSO were obtained from the NOAA Physical Sciences Laboratory website (https://psl.noaa.gov/enso/mei/). Correlations among the three weather and climate variables were all <|0.5|, allowing for all to be included in a single model. We fit a linear regression on the three weather and climate variables to determine if there is evidence of a temporal trend in them over our study period.

### 2.4 Statistical model for estimating survival

We estimated apparent annual survival (ϕ) and recapture probabilities (*p*) at both the community and the species levels by fitting a multispecies Cormack–Jolly–Seber (CJS) model using Bayesian inference (Kéry & Schaub, 2012). Apparent survival (“survival” henceforth) is defined as the probability of a marked individual to survive and be philopatric to the study site between consecutive years. The multispecies model structure allowed us to estimate survival and recapture probabilities at both the community and species‐specific levels, and we allowed for both CJS parameters to vary over time. We developed a two‐age class model (Kéry & Schaub, 2012), where individuals first captured as juveniles transition into the adult age‐class as second‐year individuals, and individuals of unknown age at banding were considered to be adults. The model considered both juveniles and adults age‐classes for thorn‐tailed Rayadito, Patagonian sierra finch, and white‐crested Elaenia because at least 25 individuals were first captured as juveniles. For the other two species an insufficient number of juveniles were first captured; hence, we focused only on estimation of adult survival for them.

### 2.5 Annual survival probabilities at the community level

This model assumes a normal distribution for logit juvenile survival $\left(\phi_{s,\mathrm{juv}}\right)$, logit adult survival $\left(\phi_{s,\mathrm{ad}}\right)$, and logit recapture probability $\left(p_{s}\right)$ for each species s:

$$\text{logit}\left(\phi_{s,\mathrm{juv}}\right)\sim \text{Normal}\left(\mu_{\phi \left(\mathrm{juv}\right)},\sigma_{\phi \left(\mathrm{juv}\right)}\right)$$

$$\text{logit}\left(\phi_{s,\mathrm{ad}}\right)\sim \text{Normal}\left(\mu_{\phi \left(\mathrm{ad}\right)},\sigma_{\phi \left(\mathrm{ad}\right)}\right)$$

$$\text{logit}\left(p_{s}\right)\sim \text{Normal}\left(\mu_{p},\sigma_{p}\right)$$

The species‐specific estimates are denoted by $\phi_{s,\mathrm{juv}}$, $\phi_{s,\mathrm{ad}}$, and *p*_s_, while the community‐level estimates are represented by the two types of hyper‐parameters $\mu_{\phi \left(\mathrm{juv}\right)}$ and $\mu_{\phi \left(\mathrm{ad}\right)}$, which are the community mean logit survival for each age class; and $\sigma_{\phi \left(\mathrm{juv}\right)}$ and $\sigma_{\phi \left(\mathrm{ad}\right)}$ are the variability of mean survival among species for each age class (all on the logit scale). The model also contains analogous recapture probability *p* hyper‐parameters.

At the community level, we explored the possible influence of migratory behavior (migrant vs. nonmigrant) and mean body size of each species on annual survival. The migratory behavior (MB) of each species at this latitude followed those described by Sandvig et al. (2020; i.e. where a species was resident or left the study area during the nonbreeding season). For the mean body size (BS) of each species, we took the mean weight from all adult individuals captured at the study size. These terms were then included in a linear regression as follows:

$$\text{logit}\left(\mu_{s,\mathrm{juv}}\right)=\text{Normal}\left(\mu_{\phi \left(\mathrm{juv}\right)}+\beta 1*{\mathrm{MB}}_{s}+\beta 2*{\mathrm{BS}}_{s},\sigma_{\phi \left(\mathrm{juv}\right)}\right)$$

$$\text{logit}\left(\mu_{s,\mathrm{ad}}\right)=\text{Normal}\left(\mu_{\phi \left(\mathrm{ad}\right)}+\beta 1*{\mathrm{MB}}_{s}+\beta 2*{\mathrm{BS}}_{s},\sigma_{\phi \left(\mathrm{ad}\right)}\right)$$

### 2.6 Annual survival probabilities at the species level

To test whether the annual survival probabilities at the species levels showed a temporal trend over the study period, we used a logit‐linear regression with a continuous year term in the model structure. In addition, the model also included terms to test for possible effects of the three climatic variables: minimum temperature, precipitation, and ENSO (see above for details on how these were calculated). For the recapture probability we assumed no differences between age classes and included an effect of catch effort (the number of hours times the area of nets deployed during each year). We also included a temporal variance term σ in each logit‐linear regression to account for unexplained temporal variation. The regressions at the species level were specified as follows:

$$\begin{array}{cc} \text{logit}\left(\phi_{t,s\left(\mathrm{juv}\right)}\right)= & \mu_{\phi \left(\mathrm{juv}\right)}+\beta 1_{s\left(\mathrm{juv}\right)}*t+\beta 2_{s\left(\mathrm{juv}\right)}*{\text{temp}}_{t}\\ & +\beta 3_{s\left(\mathrm{juv}\right)}*{\text{precip}}_{t}+\beta 4_{s\left(\mathrm{juv}\right)}*{\text{ENSO}}_{t}+\epsilon_{t,s\left(\mathrm{juv}\right)} \end{array}$$

$$\epsilon_{t,s\left(\mathrm{juv}\right)}\sim \text{Normal}\;\left(0,\sigma_{s\left(\mathrm{juv}\right)}\right)$$

$$\begin{array}{cc} \text{logit}\left(\phi_{t,s\left(\mathrm{ad}\right)}\right)= & \mu_{\phi \left(\mathrm{ad}\right)}+\beta 1_{s\left(\mathrm{ad}\right)}*t+\beta 2_{s\left(\mathrm{ad}\right)}*{\text{temp}}_{t}\\ & +\beta 3_{s\left(\mathrm{ad}\right)}*{\text{precip}}_{t}+\beta 4_{s\left(\mathrm{ad}\right)}*{\text{ENSO}}_{t}+\epsilon_{t,s\left(\mathrm{ad}\right)} \end{array}$$

$$\epsilon_{t,s\left(\mathrm{ad}\right)}\sim \text{Normal}\;\left(0,\sigma_{s\left(\mathrm{ad}\right)}\right)$$

$$\text{logit}\left(p_{t,s}\right)\sim \mu_{p}+\beta 1_{s}*{\text{effort}}_{t}+\epsilon_{t,s}$$

$$\epsilon_{t,s}\sim \text{Normal}\;\left(0,\sigma_{s}\right)$$

We placed vague priors expressed as a uniform distribution on the interval [0, 1] on the intercepts in all the regressions for ϕ and *p*. For slope parameters we chose vague priors expressed as a normal distribution with a mean of 0 and a precision of 0.1 (see Appendix S1 for a concise description of the model, including all the priors, in the BUGS language). We estimated all parameters using Markov Chain Monte Carlo (MCMC) simulation using software JAGS (Plummer, 2003). We ran three MCMC chains for 110,000 iterations, discarded the first 10,000 of them as burn‐in, and thinned the remainder by a factor of five. This resulted in 60,000 draws from the joint posterior distribution of the unknowns for inference. We checked the mixing of the three chains visually by inspecting the traceplots and confirmed convergence when *R*‐hat values were below 1.1 (Brooks & Gelman, 1998) for each parameter. All analyses were coded in R (R Core Team, 2021) and JAGS was called using the ‘jagsUI’ package (Kellner, 2021). For plotting the posterior distributions of survival estimates for each species we used the ‘denstrip’ package (Jackson, 2008) and for plotting the posterior distributions of the model fixed effects we used the ‘bayesplot’ package (Gabry & Mahr, 2021). In the paper we report parameter posterior means and 89% intervals as credible intervals (CI), as they are deemed to be more computationally stable than conventional frequentist 95% intervals in Bayesian frameworks (Kruschke, 2014). For reporting the existence of effects and significance of parameters we follow guidelines by Makowski, Ben‐Shachar, Chen, and Lüdecke (2019) using the ‘bayestestR’ package (Makowski, Ben‐Shachar, & Lüdecke, 2019). For the existence of an effect of the parameter, we report the Probability of Direction (*pd*), which varies between 50% and 100% and refers to the probability that a parameter is strictly positive or negative (described by its posterior distribution). For determining significance, we report the proportion of the Highest Density Interval (HDI) of the parameter that lies within a Region Of Practical Equivalence (ROPE), which can be considered a region of “null” values. If the HDI is completely outside the ROPE, the “null hypothesis” for this parameter is “rejected”. If the ROPE completely covers the HDI, i.e., all most credible values of a parameter are inside the ROPE, the null hypothesis is accepted. Otherwise, whether to accept or reject the null hypothesis is undecided (Makowski, Ben‐Shachar, & Lüdecke, 2019). The ROPE's lower and higher bounds were set to 0.1*SD of the response variable (all continuous variables included in the model were scaled to be equal to 1). Values <2.5% in ROPE are considered of probable significance, and values <1% in ROPE are considered significant.

Goodness‐of‐fit of the survival model was assessed for each species separately using the ‘R2ucare’ package and the capture histories summarized as m‐arrays following the guidelines from Gimenez et al. (2018). We fit an overall goodness‐of‐fit test, which tests for possible trap dependence (Test 2.CT), transient effect (Test 3.SR), and overdispersion (Test 2.Sm and Test 2.CL). For none of the species were any of these tests statistically significant, which suggested acceptable fit of our model to the data.

## 3 RESULTS

### 3.1 Capture/recapture summary

During 861 banding days across 20 years, we banded 3533 individuals of our five target species. The total number of captures and recaptures per species is reported in Table 1. Of the five species, the most frequently captured was thorn‐tailed Rayadito, and the least was austral thrush.

**TABLE 1:** Parameter estimates for five species of temperate forest passerines in the Cape Horn Biosphere Reserve, Chile.

Species	*ϕ*_j_	*ϕ*_a_	*ρ*	C/R_j_	C/R_a_
Thorn‐tailed Rayadito *Aphrastura spinicauda*	0.28 (0.04, 0.80)	0.40 (0.14, 0.72)	0.40 (0.23, 0.56)	323/36	474/86
White‐crested Elaenia *Elaenia albiceps*	0.24 (0.01, 0.81)	0.60 (0.22, 0.93)	0.11 (0.06, 0.18)	375/12	676/54
House wren *Troglodytes aedon*		0.52 (0.06, 0.93)	0.05 (0.01, 0.16)		136/4
Austral thrush *T. falcklandii*		0.45 (0.08, 0.91)	0.19 (0.05, 0.46)		73/8
Patagonian sierra finch *Phrygilus patagonicus*	0.13 (0.00, 0.82)	0.34 (0.08, 0.72)	0.16 (0.07, 0.31)	772/4	780/50

*Note*: Mean adult apparent survival (*ϕ*_a_), juvenile apparent survival (*ϕ*_j_), mean recapture probability (*p*) (posterior means and the 95% CI in parentheses for survival and recapture estimates), and number of captures and recapture (C/R). Second column gives juvenile apparent survival, followed by adult apparent survival, for three species with largest sample sizes, while for the remainder, it contains only adult apparent survival.

### 3.2 Environmental variable trends

**FIGURE 1:**
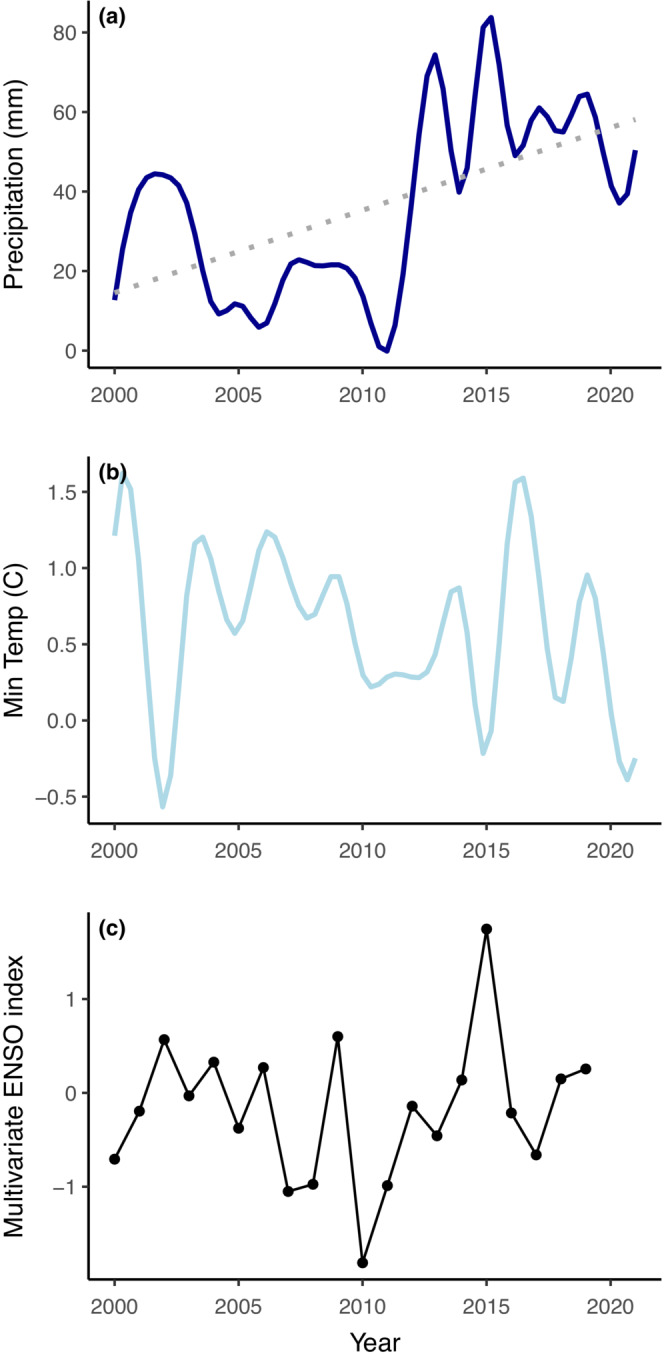
Temporal variation of climatic variables at our study site on Navarino Island, Chile (2000–2019). (a) Mean minimum daily temperature and (b) sum of precipitation during the nonbreeding season (April to August). (c) Multivariate El Niño Southern Oscillation Index, expressed as the mean of monthly values from the start of the nonbreeding season (April) until the end of the subsequent breeding season (March). Dotted gray line show a significant temporal trend in precipitation.

We found a significant positive temporal trend in minimum temperature during winter months over our study period (*β* = 0.10, *p* value = 0.005). No temporal trend was found for precipitation or ENSO (Figure 1).

### 3.3 Apparent survival probabilities

**FIGURE 2:**
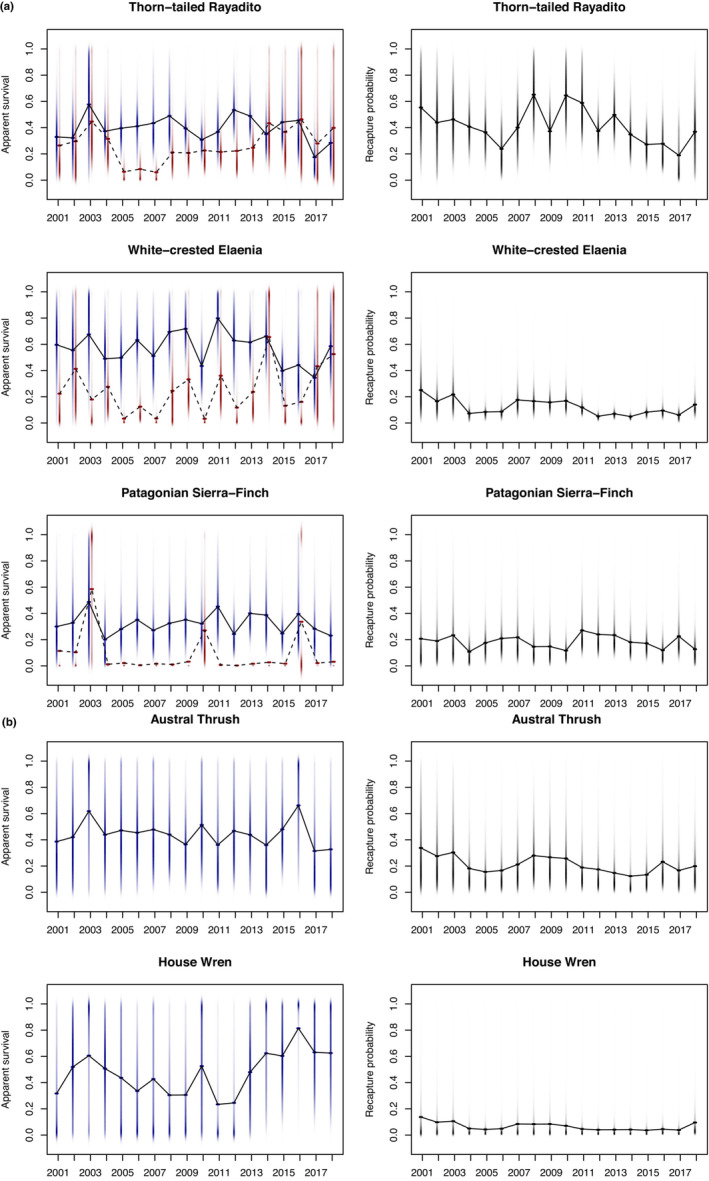
(a, b) Annual apparent survival and recapture probability estimates for five species of sub‐Antarctic Forest birds at the Omora Ethnobotanical Park, Chile, between 2000 and 2019. Colored strips show posterior distribution for each year's estimate (adults = dark blue; juveniles = dark red). The lines show the posterior mean apparent survival from year to year (adults = solid line; juveniles = dashed line) and mean recapture probability for both age classes (solid line). Vertical shaded strips depict the posterior distribution of annual recapture estimates with the darkness proportional to the probability density.

Mean juvenile annual apparent survival probabilities at the species level (Table 1; Figure 2) ranged from 0.13 (Patagonian sierra finch, *P. patagonicus*) to 0.28 (thorn‐tailed Rayadito, *A. spinicauda*), with a mean of 0.22 among the three species. Mean adult apparent survival ranged from 0.36 (Patagonian sierra finch, *P. patagonicus*) to 0.60 (white‐crested Elaenia, *E. albiceps*), with a mean of 0.47 among the five species (Table 1). Recapture probabilities was the lowest for house wren (0.05) and the highest for thorn‐tailed Rayadito (0.40; Table 1), with a mean of 0.20 among the five species of the community. Temporal variations in survival and recapture probabilities for each species are shown in Figure 2.

### 3.4 Community‐ and species‐level effects

At the community level, we found no evidently significant association between body size or MB and survival probabilities among the five species in either age class (See Supporting Information).

**FIGURE 3:**
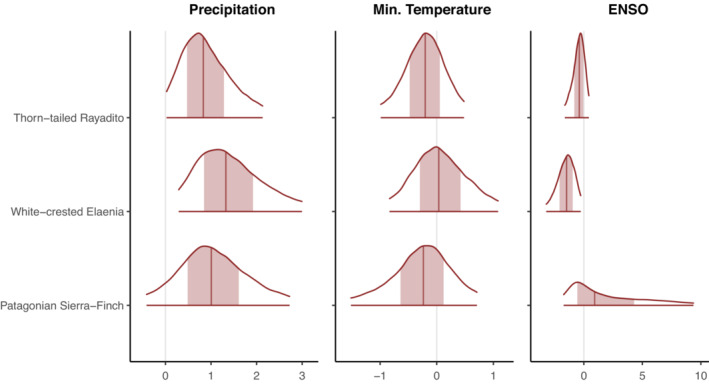
Posterior distributions of species‐level covariate effects on survival estimates for juveniles of three species of sub‐Antarctic Forest birds Sum of precipitation during the nonbreeding season; mean minimum daily temperature during the nonbreeding season; multivariate El Niño Southern Oscillation Index. Plots show the 89% CI of the posterior distribution, shaded areas represent the 50% interval, and the solid vertical line is the posterior mean.

**FIGURE 4:**
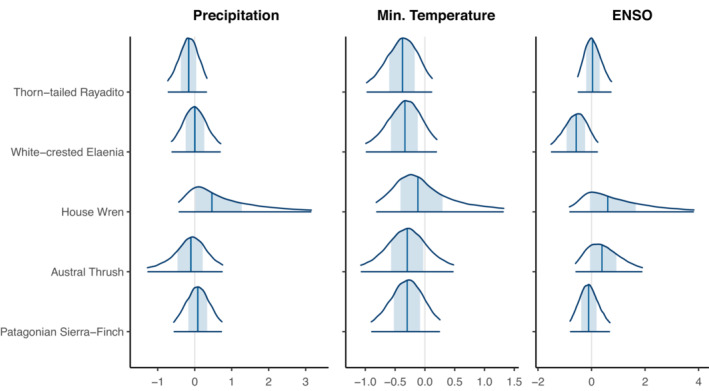
Posterior distributions of species‐level covariate effects on survival estimates for adults of five species of sub‐Antarctic Forest birds. Sum of precipitation during the nonbreeding season; mean minimum daily temperature during the nonbreeding season; Multivariate El Niño Southern Oscillation Index. Plots show the 89% CI of the posterior distribution, shaded areas represent the 50% interval, and the solid vertical line is the posterior mean.

We found support for both a significant positive effect of precipitation (mean = 1.45, *pd* = 97.98%, 89% CI [0.15, 2.78], 0.34% in ROPE) and a probable negative effect of ENSO (mean = −1.60, *pd* = 97.19%, 89% CI [−3.03, −0.16], 1.19% in ROPE) on juvenile survival of the white‐crested Elaenia (Figure 3). No evidence was found of other significant associations between climatic variables and variation in survival probabilities of juveniles or adults. However, it is worth noting that the probability of direction of winter precipitation on juvenile survival was consistently positive, and near‐significance thresholds (*pd =* 90%–98%) in the three species and for adult survival minimum winter temperature was negative (*pd* = 77%–89%) in four of the five species (Figure 4). All effect indices can be found in Supporting Information.

Regarding the temporal variations in survival probabilities, we did not find any temporal trends for juveniles or adults for any of the nine species (Figure S2).

### 3.5 Latitudinal comparison

When comparing the survival estimates from this study with those from studies at lower latitudes in the same species, we found that adult apparent survival was higher at our high‐latitude site in all cases (Table 2).

**TABLE 2:** Intraspecific comparison of survival estimates from this study with those found from other studies at lower latitudes in South America.

Species	This study 54°S	Lower‐latitude study sites	References
Thorn‐tailed Rayadito *Aphrastura spinicauda*	0.40 (0.14, 0.72)	0.68 (0.64–0.72) 30°S 0.65 (0.57–0.73) 30°S	Botero‐Delgadillo et al. (2017), Quirici et al. (2021)
White‐crested Elaenia *Elaenia albiceps*	0.60 (0.22, 0.93)	0.61 (0.34–0.95) 41°S	Presti et al. (2018)
Austral thrush *T. falcklandii*	0.45 (0.08, 0.91)	0.83^a^ 39°S	Ricklefs (1997)

^a^ Ricklefs (1997) did not give a measure of error for the survival estimate.

## 4 DISCUSSION

Our findings provide important information on survival probabilities of an understudied forest bird community in the southern cone of South America. Among the examined species, precipitation and ENSO were found to affect survival in juveniles of a migratory species (white‐crested Elaenia), but we did not find strong evidence of effects of these variables on survival in adults. On the other hand, although we did not find strong evidence of an effect of minimum temperature during winter on survival, we found a consistent positive effect on all juvenile estimates and four out of five adults. At the community level, body size and MB were not good predictors of differences in survival among the examined species. Additionally, when comparing survival estimates from species in this community with counterparts at lower latitudes, we found a decreasing latitudinal gradient, as do previous studies comparing survival at different latitudes in the Northern Hemisphere (see Scholer et al., 2020).

### 4.1 Community‐ and species‐level effects

Allometric scaling of survival with body size in birds is a widespread and well‐documented pattern (Maness & Anderson, 2013; Sæther, 1989; Scholer et al., 2020). From our model, no clear pattern between survival and body size emerges for adults or juveniles at our study site; however, the inability to detect this interspecific pattern may be simply due to the low number of species we were able to include. Scholer et al.'s (2020) global meta‐analysis of survival rates found resident species have higher survival than migrants. Likewise, as with body size, the handful of species we were able to include in the analysis was probably not enough to detect interspecific patterns between migrant and nonmigrant species. Having sufficient data for a larger number of species will be imperative to properly investigate these community‐level patterns and address whether they are consistent with patterns found at the global scale.

At the species level, we found differing effects between age classes, with winter precipitation affecting juvenile survival in one out of three species. However, we did not find these effects on adult survival in the same species. Precipitation can sometimes have positive indirect effects on juvenile survival through increased food availability in the breeding season (Dybala, Eadie, et al., 2013), which may be the case for juveniles of white‐crested Elaenia, as juveniles of this migratory species would only experience the effects of winter precipitation indirectly once having returned to the breeding ground. We did not find strong evidence of an effect of minimum temperature during winter on survival probabilities in either juveniles or adults. However, we did find a consistent positive effect on all juvenile estimates and four out of five in adults. From these results, it is difficult to elucidate if there is indeed a positive effect of minimum winter temperature on survival in these species, which could be resolved with a larger amount of data.

On the other hand, we found a probable negative effect of ENSO on juvenile survival in the single long‐distance migrant species of the group (white‐crested Elaenia), suggesting that in this case the effect would be linked to the conditions experienced during migration or on the nonbreeding range of this species. McKellar et al. (2015) found that a migratory species, American Redstart (*Setophaga ruticilla*), from the Northern Hemisphere experienced lower survival during La Niña and El Niño conditions. In the case of high‐latitude populations of white‐crested Elaenia, which migrate to northern Brazil for the nonbreeding season (Jiménez et al., 2016), El Niño events lead to drought conditions in their wintering region (Ropelewski & Halpert, 1987) and the Patagonia region (Paruelo et al., 1998) during their migration. This could explain the negative association we found between survival and ENSO, as negative values of this index are related to El Niño conditions. These effects of climatic variables on the survival of some species raises the question of what we may expect for populations under future climatic conditions.

Estimates of survival from our model were substantially lower in juveniles than in adults for the three species examined at both age classes. Among the three species, juvenile Patagonian sierra finches showed extremely low interannual survival, with several yearly estimates being indistinguishable from zero. This likely due to the low number of juveniles recaptured the subsequent year in the years of estimates close to zero, which may be related to low interannual site fidelity of juveniles after their initial dispersal; however, no information is available about dispersal in this species. Furthermore, the goodness‐of‐fit test did not show evidence indicative of a transient effect. The highest survival for any species was estimated for the thorn‐tailed Rayadito, one of the most abundant species in these forests (Anderson & Rozzi, 2000; Ippi et al., 2009). This species also had by far the highest recapture rate, suggesting high interannual site fidelity.

Recent climate change at high latitudes of the Northern Hemisphere have manifested in increased mean temperatures (Arias et al., 2021). However, at our Southern Hemisphere high‐latitude study site, we did not find a significant temporal trend in mean minimum temperatures during winter over the study period. We did, nonetheless, detect an increase in the accumulated precipitation during winter months across our study period. Although we found evidence that precipitation was related to juvenile survival in one species, but in none for adult survival, increased precipitation could pose contrasting effects on survival between these two age classes in this community. Dybala, Eadie, et al. (2013) found that juvenile survival in a small songbird of North America increased with rainy season precipitation, up to a threshold, where it then decreased; while adult survival decreased with precipitation, posing contrasting implications for population growth rates. Furthermore, if high precipitation is also experienced during the breeding season, this can have detrimental effects on nestling survival (Sandvig et al., 2017). The CHBR presents one of the most extreme gradients of annual precipitation globally, ranging from >5000 to <500 mm (Aguirre et al., 2021). In this context, high annual precipitation has been related to reduced species richness in these subantarctic forests (Quilodrán et al., 2022), suggesting some type of precipitation threshold for the subsistence of some species. Future climate projections for Chile in the coming decades predict warmer minimum temperatures and an increase in accumulated precipitation at high latitudes (Araya‐Osses et al., 2020). According to the relationships derived from our model, these projected changes in temperature could possibly have a positive effect on most species of our study in both age classes, while precipitation could have a positive effect on juvenile survival in one of the species of this community, but possible negative effects if accumulated precipitations exceed a certain threshold. Future studies should focus on improving our understanding of the relationship between climate and reproduction to provide a more complete picture of the demographic processes experienced in these subantarctic forests, allowing full demographic models to project potential impacts of climate change.

### 4.2 Latitudinal comparisons

Our estimates allowed us to compare survival between our site and those of previous studies at lower latitudes, covering a large latitudinal range in South America. For the three species we found conspecific estimates of survival in the literature, survival rates were lower at higher latitudes, following the same pattern reported for the Northern Hemisphere by Scholer et al. (2020). Additionally, in a study in Ecuador (0°37′S; Blake & Loiselle, 2013), survival rates were reported for a group of species in the same families as those we report for Navarino Island. Among four families, our findings agree with the ones found in the Northern Hemisphere (Pizarro Muñoz et al., 2018; Scholer et al., 2020), with the mean adult survival estimates in Ecuador (Furnariidae, 0.61 (*n* = 7); Tyrannidae, 0.61 (*n* = 4); Troglodytidae, 0.66 (*n* = 2); Turdidae, 0.66 (*n* = 1)) being higher than for the same families on Navarino Island (Furnariidae, 0.31 (*n* = 1); Tyrannidae, 0.29 (*n* = 1); Troglodytidae, 0.52 (*n* = 1); and Turdidae, 0.45 (*n* = 1)). Although our comparison only comprises a small number of species, it suggests that survival rates in the southern cone follow the same pattern reported for species in other regions of the globe.

## 5 CONCLUSIONS

The survival estimates we provide in this study are a key resource for predicting population changes in the forest bird community under future climate change scenarios in this unique region of the world. Our results highlight the importance of considering differences in the sensitivities to weather between different age classes and species, as we have documented here that they are not equal throughout the community. Despite temperature often being considered the most important variable related to climate change, we show that variations in precipitation may be more impactful in some communities such as this one in the Magellanic sub‐Antarctic Forest. Finally, the long‐term collection of mark–recapture data is necessary for modeling detailed demographic processes that span a wide breath of temporal variations in climatic conditions. This work at the remote southern tip of South America showcases the importance of LTER projects in often underrepresented areas of the globe.

## AUTHOR CONTRIBUTIONS

**Erik M. Sandvig:** Conceptualization (lead); data curation (lead); formal analysis (lead); methodology (lead); visualization (lead); writing – original draft (lead); writing – review and editing (lead). **Claudio S. Quilodran:** Writing – original draft (supporting); writing – review and editing (equal). **Tomás A. Altamirano:** Writing – original draft (supporting); writing – review and editing (equal). **Francisco Aguirre:** Data curation (supporting). **Omar Barroso:** Data curation (equal). **Juan Rivero de Aguilar:** Data curation (equal); writing – review and editing (supporting). **Michael Schaub:** Formal analysis (equal); methodology (equal); supervision (lead); writing – original draft (supporting); writing – review and editing (supporting). **Marc Kery:** Formal analysis (equal); methodology (equal); supervision (lead); writing – original draft (supporting); writing – review and editing (supporting). **Rodrigo A. Vasquez:** Funding acquisition (equal); supervision (equal); writing – review and editing (supporting). **Ricardo Rozzi:** Funding acquisition (equal); supervision (supporting); writing – review and editing (supporting).

### OPEN RESEARCH BADGES

This article has earned an Open Data badge for making publicly available the digitally‐shareable data necessary to reproduce the reported results. The data is available at https://github.com/Chilesummits/survival‐in‐the‐world‐s‐southernmost‐forests.

## Supporting information


Data S1

## Data Availability

The data that support the findings of this study are openly available at https://github.com/Chilesummits/survival‐in‐the‐world‐s‐southernmost‐forests. DOI 10.6084/m9.figshare.22819832.
