# Supplementary material for: Survival rates in the world's southernmost forest bird community
Source: Ecol Evol. 2023 Jun 20;13(6):e10143. doi: 10.1002/ece3.10143 (PMC10282503; doi:10.1002/ece3.10143)
Supplement: Supplementary file 1 — Data S1 [file ECE3-13-e10143-s001.docx]

**Supporting Information**

**Figure S1**. Posterior distributions of community-level covariate effects for mean body size (in grams) and migratory behavior (migrants and nonmigrants) on yearly survival estimates for juveniles (A) and adults (B) for a group of five species of sub-Antarctic Forest birds. Plots show the 89% credible interval of the posterior distribution, shaded areas represent the 50% interval and the solid vertical line is the posterior mean

**Table S1.** Parameter estimates for community-level covariate effects of mean body size and migratory behavior on yearly survival estimates for juveniles and adults of a group of 5 species of sub-Antarctic Forest birds. ROPE [-0.1*SD, 0.1*SD]

|  | **Parameter** | **Mean** | **89% CI** | **pd** | **% in ROPE** |
| --- | --- | --- | --- | --- | --- |
| Juveniles | Body Size | -0.71 | -1.96, 0.48 | 83.42% | 6.64% |
|  | Migrant | 0.20 | -2.28, 2.61 | 55.41% | 5.83% |
|  | Nonmigrant | -0.06 | -2.40, 2.33 | 51.49% | 6.12% |
| Adults | Body Size | 0.05 | -0.64, 0.74 | 54.62% | 20.84% |
|  | Migrant | 0.15 | -1.65, 1.93 | 56.04% | 7.76% |
|  | Nonmigrant | -0.51 | [-2.39, 1.39] | 67.72% | 6.80% |

**Table S2.** Parameter estimates for species-level covariate effects of accumulated winter precipitation, minimum winter temperature and ENSO on annual juvenile apparent survival estimates for three species of sub-Antarctic Forest birds. ROPE [-0.1*SD, 0.1*SD]

|  | **Species** | **Mean** | **89% CI** | **pd** | **% in ROPE** |
| --- | --- | --- | --- | --- | --- |
| Precip | Thorn-tailed Rayadito | 0.93 | -0.10, 1.95 | 94.99% | 4.29% |
|  | White-crested Elaenia | 1.45 | 0.15, 2.78 | **97.98%** | **0.34%** |
|  | Patagonian Sierra-Finch | 1.07 | -0.48, 2.65 | 88.95% | 3.93% |
| Min. Temp | Thorn-tailed Rayadito | -0.22 | -0.96, 0.50] | 70.41% | 18.83% |
|  | White-crested Elaenia | 0.07 | -0.90, 1.01 | 53.02% | 16.57% |
|  | Patagonian Sierra-Finch | -0.30 | -1.39, 0.80 | 67.22% | 14.48% |
| ENSO | Thorn-tailed Rayadito | -0.47 | -1.52, 0.50 | 77.61% | 12.54% |
|  | White-crested Elaenia | -1.60 | -3.03, -0.16 | **97.19%** | **1.19%** |
|  | Patagonian Sierra-Finch | 2.21 | -2.36, 7.88 | 63.70% | 3.85% |

**Table S3.** Parameter estimates for species-level covariate effects of accumulated winter precipitation, minimum winter temperature and ENSO on annual adult apparent survival estimates for five species of sub-Antarctic Forest birds. ROPE [-0.1*SD, 0.1*SD]

|  | **Species** | **Mean** | **89% CI** | **pd** | **% in ROPE** |
| --- | --- | --- | --- | --- | --- |
| Precip | Thorn-tailed Rayadito | -0.18 | -0.72, 0.34 | 70.50% | 23.71% |
|  | White-crested Elaenia | 0.02 | -0.67, 0.66 | 50.29% | 22.52% |
|  | House Wren | 0.83 | -0.69, 2.48 | 76.11% | 12.31% |
|  | Austral Thrush | -0.17 | -1.17, 0.84 | 58.74% | 17.22% |
|  | Patagonian Sierra-Finch | 0.08 | -0.56, 0.74 | 58.82% | 21.82% |
| Min. Temp | Thorn-tailed Rayadito | -0.40 | -0.94, 0.16 | 89.21% | 12.42% |
|  | White-crested Elaenia | -0.36 | -0.95, 0.24 | 85.08% | 14.55% |
|  | House Wren | 0.02 | -0.98, 1.07 | 59.46% | 14.97% |
|  | Austral Thrush | -0.30 | -1.09, 0.47 | 76.98% | 14.44% |
|  | Patagonian Sierra-Finch | -0.31 | -0.88, 0.27 | 82.11% | 16.38% |
| ENSO | Thorn-tailed Rayadito | 0.07 | -0.56, 0.69 | 54.44% | 22.88% |
|  | White-crested Elaenia | -0.60 | -1.46, 0.28 | 87.66% | 8.45% |
|  | House Wren | 0.96 | -1.09, 3.37 | 72.62% | 8.68% |
|  | Austral Thrush | 0.49 | -0.74, 1.71 | 72.05% | 11.55% |
|  | Patagonian Sierra-Finch | -0.09 | -0.85, 0.64 | 60.18% | 18.82% |

**Figure S2**. Posterior distributions of species-level covariate effects for temporal trends in survival for a) juveniles in three species and b) adults in five species over the study period. Plots show the 89% credible interval of the posterior distribution, shaded areas represent the 50% interval and the solid vertical line is the posterior mean.

**Table S4.** Posterior distributions of species-level covariate effects for temporal trends in survival for a) juveniles and b) adults over the study period of a group of 5 species of sub-Antarctic Forest birds. ROPE [-0.1*SD, 0.1*SD]

|  | **Species** | **Mean** | **89% CI** | **pd** | **% in ROPE** |
| --- | --- | --- | --- | --- | --- |
| Juveniles | Thorn-tailed Rayadito | -0.02 | -0.20, 0.15 | 56.27% | 71.61% |
|  | White-crested Elaenia | -0.05 | -0.27, 0.16 | 66.05% | 57.87% |
|  | Patagonian Sierra-Finch | -0.32 | -0.87, 0.17 | 84.41% | 31.28% |
| Adults | Thorn-tailed Rayadito | -7.92e-03 | -0.10, 0.09 | 57.32% | 95.20% |
|  | White-crested Elaenia | -0.02 | -0.15, 0.09 | 62.38% | 84.82% |
|  | House Wren | 0.01 | -0.13, 0.15 | 50.45% | 82.57% |
|  | Austral Thrush | -5.37e-03 | -0.14, 0.13 | 54.77% | 83.37% |
|  | Patagonian Sierra-Finch | -0.02 | -0.12, 0.09 | 60.88% | 90.87% |

MODEL CODE

# Multi-species model in BUGS language

model {

# Survival regressions with three climate covariate effects plus

# one set of parameters to estimate temporal trend in survival

# These are the basic species-level linear models for juv and ad phi

# Both regressions contain random year effects

# Juvenile and adults are separated because there are only 3 species with juvenile data and all 9 with adult data

for (s in 1:(nspecies.j)){

for (t in 1:(nyears-1)){

logit(phi.j[t,s]) <- mu.j[s] + beta.j1[s]*precip[t] + beta.j2[s]*min.temp[t] + beta.j3[s]*enso[t] + beta.j.trend[s]*t + epsilon.js[t,s]

}

}

for (s in 1:(nspecies)){

for (t in 1:(nyears-1)){

logit(phi.a[t,s]) <- mu.a[s] + beta.a1[s]*precip[t] + beta.a2[s]*min.temp[t] + beta.a3[s]*enso[t] + beta.a.trend[s]*t + epsilon.as[t,s]

}

}

# Recapture rates dependent on net effort (same for both ages)

# And this is the linear model for recapture

# It also has random year effects

for (s in 1:(nspecies)){

for (t in 1:(nyears-1)){

logit(p[t,s]) <- mu.p[s] + beta.p[s]*net.eff[t] + epsilon.p[t,s] #Recapture is dependent on net effort

}

}

# Priors and community structure

# Species-specific annual random effects (= 'annual residuals')

# in the two survival rates and in recapture

for (s in 1:(nspecies.j)){

for (t in 1:(nyears-1)){

epsilon.js[t,s] ~ dnorm(0, tau.j[s])

}

}

for (s in 1:(nspecies)){

for (t in 1:(nyears-1)){

epsilon.as[t,s] ~ dnorm(0, tau.a[s])

epsilon.p[t,s] ~ dnorm(0, tau.p[s])

}

}

# Priors for yearly random-effects for juv and ad survival

for (s in 1:(nspecies.j)){

tau.j[s] <- 1 / sigma2.j[s]

log(sigma2.j[s]) <- lsigma2.j[s]

lsigma2.j[s] ~ dnorm(mu.lsigma2.j, tau.lsigma2.j)

sigma.j[s] <- sqrt(sigma2.j[s])

}

for (s in 1:(nspecies)){

tau.a[s] <- 1 / sigma2.a[s]

log(sigma2.a[s]) <- lsigma2.a[s]

lsigma2.a[s] ~ dnorm(mu.lsigma2.a, tau.lsigma2.a)

sigma.a[s] <- sqrt(sigma2.a[s])

}

# Community models for survival

# These relate the species- and the community-level parameters

for (s in 1:(nspecies.j)){

# Community model (prior) for the two sets of intercepts

# (for juv)

mean.phi.j[s] <- ilogit(mu.j[s])

mu.j[s] ~ dnorm(mu.mu.j[s], tau.mu.j)

# Here now we add the linear regression on migration and body masses

mu.mu.j[s] <- alpha.mu.mu.j[migration[s]] + beta.mu.mu.j * lbm.juv[s]

# Community models (priors) for 4 slope params in juvenile survival

beta.j1[s] ~ dnorm(mu.beta.j1, tau.beta.mu.j1)

beta.j2[s] ~ dnorm(mu.beta.j2, tau.beta.mu.j2)

beta.j3[s] ~ dnorm(mu.beta.j3, tau.beta.mu.j3)

beta.j.trend[s] ~ dnorm(mu.beta.j.t, tau.beta.mu.j.t)

}

for (s in 1:(nspecies)){

# Community model (prior) for the two sets of intercepts

# for ad survival

mean.phi.a[s] <- ilogit(mu.a[s])

mu.a[s] ~ dnorm(mu.mu.a[s], tau.mu.a)

# Here we add the linear regression on migration and body masses

mu.mu.a[s] <- alpha.mu.mu.a[migration[s]] + beta.mu.mu.a * lbm.ad[s]

# Community models (priors) for 4 slope params in adult survival

beta.a1[s] ~ dnorm(mu.beta.a1, tau.beta.mu.j1)

beta.a2[s] ~ dnorm(mu.beta.a2, tau.beta.mu.j2)

beta.a3[s] ~ dnorm(mu.beta.a3, tau.beta.mu.j3)

beta.a.trend[s] ~ dnorm(mu.beta.a.t, tau.beta.mu.a.t)

}

# Priors for hyperparameters

# For the species-level intercept community model

for(k in 1:2){

alpha.mu.mu.j[k] <- logit(gm.mu.j[k])

gm.mu.j[k] ~ dunif(0, 1)

}

beta.mu.mu.j ~ dnorm(0, 0.1)

tau.mu.j <- pow(sd.mu.j, -2)

sd.mu.j ~ dunif(0, 5)

for(k in 1:2){

alpha.mu.mu.a[k] <- logit(gm.mu.a[k])

gm.mu.a[k] ~ dunif(0,1)

}

beta.mu.mu.a ~ dnorm(0, 0.1)

tau.mu.a <- pow(sd.mu.a, -2)

sd.mu.a ~ dunif(0, 5)

# For the species-level slopes in the regressions

# juv

mu.beta.j1 ~ dnorm(0,0.1)

tau.beta.mu.j1 <- pow(sd.beta.mu.j1, -2)

sd.beta.mu.j1 ~ dunif(0,10)

mu.beta.j2 ~ dnorm(0,0.1)

tau.beta.mu.j2 <- pow(sd.beta.mu.j2, -2)

sd.beta.mu.j2 ~ dunif(0,10)

mu.beta.j3 ~ dnorm(0,0.1)

tau.beta.mu.j3 <- pow(sd.beta.mu.j3, -2)

sd.beta.mu.j3 ~ dunif(0,10)

mu.beta.j.t ~ dnorm(0,0.1)

tau.beta.mu.j.t <- pow(sd.beta.mu.j.t, -2)

sd.beta.mu.j.t ~ dunif(0,2)

# ad

mu.beta.a1 ~ dnorm(0,0.1)

tau.beta.mu.a1 <- pow(sd.beta.mu.a1, -2)

sd.beta.mu.a1 ~ dunif(0,10) # increased to 10 because previous runs 95% quantiles were very close to 2

mu.beta.a2 ~ dnorm(0,0.1)

tau.beta.mu.a2 <- pow(sd.beta.mu.a2, -2)

sd.beta.mu.a2 ~ dunif(0,10) # increased to 10 because previous runs 95% quantiles were very close to 2

mu.beta.a3 ~ dnorm(0,0.1)

tau.beta.mu.a3 <- pow(sd.beta.mu.a3, -2)

sd.beta.mu.a3 ~ dunif(0,10) # increased to 10 because previous runs 95% quantiles were very close to 2

mu.beta.a.t ~ dnorm(0,0.1)

tau.beta.mu.a.t <- pow(sd.beta.mu.a.t, -2)

sd.beta.mu.a.t ~ dunif(0,2)

# Variances

# juv

mu.lsigma2.j ~ dnorm(0,0.01)

tau.lsigma2.j <- pow(sd.lsigma2.j, -2)

sd.lsigma2.j ~ dunif(0,2)

# ad

mu.lsigma2.a ~ dnorm(0,0.01)

tau.lsigma2.a <- pow(sd.lsigma2.a, -2)

sd.lsigma2.a ~ dunif(0,2)

# Community models for recapture

for (s in 1:(nspecies)){

mean.p[s] <- ilogit(mu.p[s]) # logit transformation

mu.p[s] ~ dnorm(mu.mu.p, tau.mu.p)# prior for logit of mean recapture

beta.p[s] ~ dnorm(mu.beta.p, tau.beta.mu.p) # Prior for slope parameter

tau.p[s] <- 1 / sigma2.p[s]

log(sigma2.p[s]) <- lsigma2.p[s]

lsigma2.p[s] ~ dnorm(mu.lsigma2.p, tau.lsigma2.p)

sigma.p[s] <- sqrt(sigma2.p[s])

}

# Priors for recapture hyperparameters

# For the species-level intercept community model

mu.mu.p <- logit(gm.mu.p)

gm.mu.p ~ dunif(0, 1)

tau.mu.p <- pow(sd.mu.p, -2)

sd.mu.p ~ dunif(0, 5)

# For the species-level slope community model

mu.beta.p ~ dnorm(0,0.1)

tau.beta.mu.p <- pow(sd.beta.mu.p, -2)

sd.beta.mu.p ~ dunif(0,2)

# Recapture variances

mu.lsigma2.p ~ dnorm(0,0.01)

tau.lsigma2.p <- pow(sd.lsigma2.p, -2)

sd.lsigma2.p ~ dunif(0,2)

# Define the multinomial likelihood for the counts in the 2 m-arrays

# 1. For the data sets where there are juv and adults

for (s in 1:(nspecies.j)){

for (t in 1:(nyears-1)){

marr.j[t,1:nyears,s] ~ dmulti(pr.j[t,,s], rel.j[t,s])

marr.a[t,1:nyears,s] ~ dmulti(pr.a[t,,s], rel.a[t,s])

}

}

# Define the cell probabilities of the m-arrays

# Main diagonal

for (s in 1:(nspecies.j)){

for (t in 1:(nyears-1)){

q[t,s] <- 1-p[t,s] # Probability of non-recapture

pr.j[t,t,s] <- phi.j[t,s]*p[t,s]

pr.a[t,t,s] <- phi.a[t,s]*p[t,s]

# Above main diagonal

for (j in (t+1):(nyears-1)){

pr.j[t,j,s] <- phi.j[t,s]*prod(phi.a[(t+1):j,s])*prod(q[t:(j-1),s])*p[j,s]

pr.a[t,j,s] <- prod(phi.a[t:j,s])*prod(q[t:(j-1),s])*p[j,s]

} #j

# Below main diagonal

for (j in 1:(t-1)){

pr.j[t,j,s] <- 0

pr.a[t,j,s] <- 0

} #j

} #t

}

# Last column: probability of non-recapture

for (s in 1:(nspecies.j)){

for (t in 1:(nyears-1)){

pr.j[t,nyears,s] <- 1-sum(pr.j[t,1:(nyears-1),s])

pr.a[t,nyears,s] <- 1-sum(pr.a[t,1:(nyears-1),s])

} #t

}

# 2. for the data sets where there is adults only

for (s in 4:(nspecies)){

for (t in 1:(nyears-1)){

marr.a[t,1:nyears,s] ~ dmulti(pr.a[t,,s], rel.a[t,s])

}

}

# Define the cell probabilities of the m-arrays

# Main diagonal

for (s in 4:(nspecies)){

for (t in 1:(nyears-1)){

q[t,s] <- 1-p[t,s] # Probability of non-recapture

pr.a[t,t,s] <- phi.a[t,s]*p[t,s]

# Above main diagonal

for (j in (t+1):(nyears-1)){

pr.a[t,j,s] <- prod(phi.a[t:j,s])*prod(q[t:(j-1),s])*p[j,s]

} #j

# Below main diagonal

for (j in 1:(t-1)){

pr.a[t,j,s] <- 0

} #j

} #t

}

# Last column: probability of non-recapture

for (s in 4:(nspecies)){

for (t in 1:(nyears-1)){

pr.a[t,nyears,s] <- 1-sum(pr.a[t,1:(nyears-1),s])

} #t

}

}
